# Supplementary figures and images for: T-Cell Hyperactivation and Paralysis in Severe COVID-19 Infection Revealed by Single-Cell Analysis
Source: Front Immunol. 2020 Oct 8;11:589380. doi: 10.3389/fimmu.2020.589380 (PMC7596772; doi:10.3389/fimmu.2020.589380)

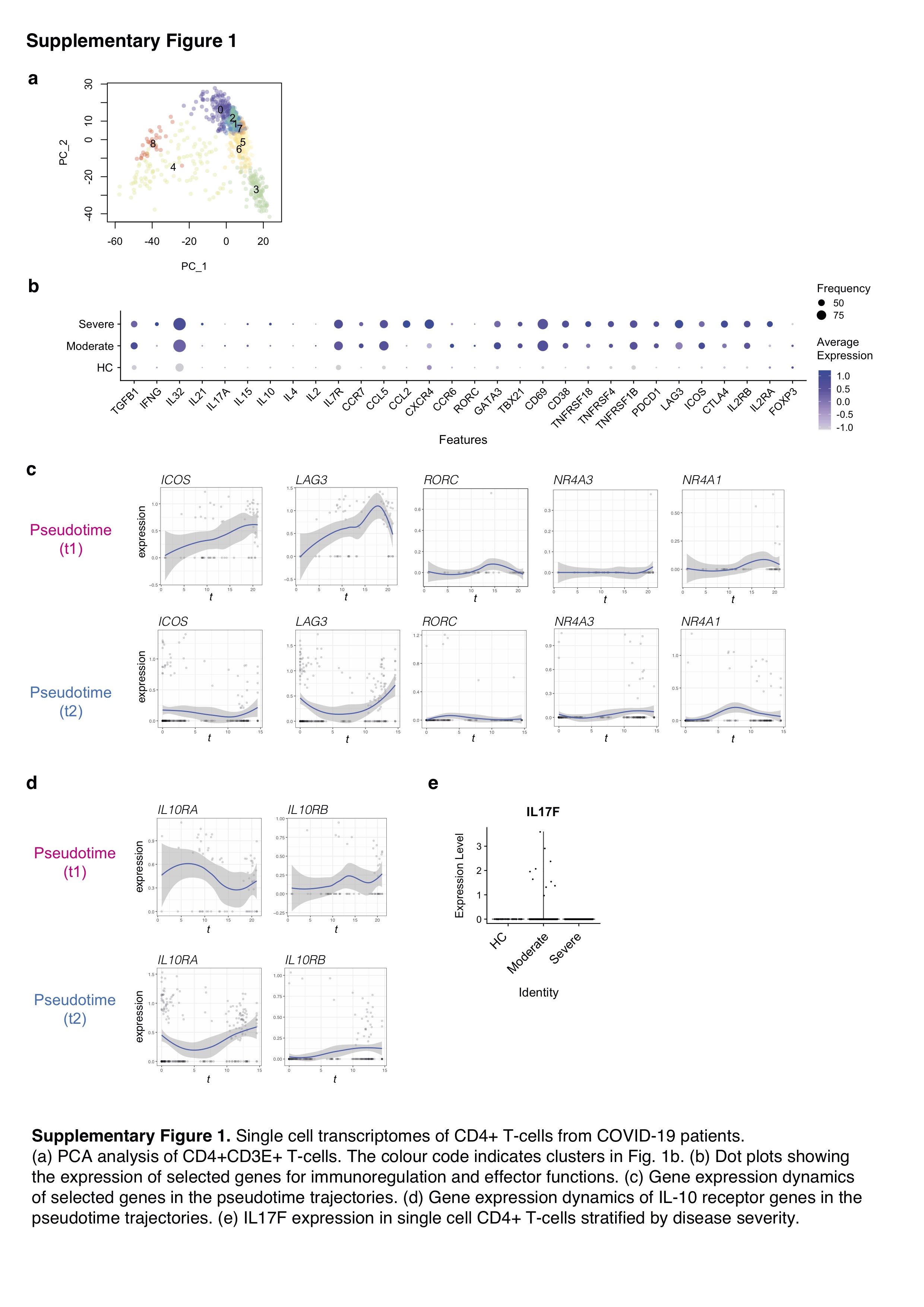

Supplement: Supplementary file 1 [file Image_1.jpg]
